# Review and Evaluation of Eye Movement Event Detection Algorithms

**DOI:** 10.3390/s22228810

**Published:** 2022-11-15

**Authors:** Birtukan Birawo, Pawel Kasprowski

**Affiliations:** Department of Applied Informatics, Silesian University of Technology, Akademicka 16, 44-100 Gliwice, Poland

**Keywords:** eye tracking, eye movement events, fixations, saccades, event detection algorithms

## Abstract

Eye tracking is a technology aimed at understanding the direction of the human gaze. Event detection is a process of detecting and classifying eye movements that are divided into several types. Nowadays, event detection is almost exclusively done by applying a detection algorithm to the raw recorded eye-tracking data. However, due to the lack of a standard procedure for how to perform evaluations, evaluating and comparing various detection algorithms in eye-tracking signals is very challenging. In this paper, we used data from a high-speed eye-tracker SMI HiSpeed 1250 system and compared event detection performance. The evaluation focused on fixations, saccades and post-saccadic oscillation classification. It used sample-by-sample comparisons to compare the algorithms and inter-agreement between algorithms and human coders. The impact of varying threshold values on threshold-based algorithms was examined and the optimum threshold values were determined. This evaluation differed from previous evaluations by using the same dataset to evaluate the event detection algorithms and human coders. We evaluated and compared the different algorithms from threshold-based, machine learning-based and deep learning event detection algorithms. The evaluation results show that all methods perform well for fixation and saccade detection; however, there are substantial differences in classification results. Generally, CNN (Convolutional Neural Network) and RF (Random Forest) algorithms outperform threshold-based methods.

## 1. Introduction

Eye tracking is the process of tracking the movement of the eyes to know exactly where and for how long a person is looking [1]. The primary purpose of eye movement is to direct the eyes towards the targeted object and keep it at the center of the fovea to provide a clear vision of the object. Eye tracking is used in various research fields such as cognitive science, psychology, neurology, engineering, medicine and marketing, to mention some [2]. Human–computer interaction is another example of applications—it is beneficial for disabled people to interact with a computer through gaze [3]. Eye tracking can also be used to monitor and control automobile drivers [4]. It is thus highly interdisciplinary and used in various fields, which is also reflected in how eye-tracking hardware and software have been developed over the years [5]. To extract useful information, the raw eye movements are typically converted into so-called events. This process is named event detection.

The goal of eye movement event detection in eye-tracking research is to extract events, such as fixations, saccades, post-saccadic oscillations, smooth pursuits from the stream of raw eye movement data on a set of basic criteria and rules which are appropriate for the recorded data. This classification of recorded raw eye-tracking data into events is based on some assumptions about fixation durations, saccadic amplitudes and saccadic velocities [6]. Classifying raw eye-tracker data into eye movement events reduces the complexity of eye movement analysis [7]. The classification may be done by algorithms that are considered more objective, faster and more reproducible than manual human coding.

The event detection procedure in eye tracking is associated with many challenges. One of these is that many different types of disturbances and noises may occur in the recorded signal, which originates from the individual differences among the users and eye trackers. This variability between individuals and signal qualities may create signals that are difficult for analysis. Therefore, the challenge is to develop robust algorithms that are flexible enough to be used for signals with different types of events and disturbances and that can handle different types of eye trackers and different individuals. Another challenge of eye movement event detection in eye tracking signals is evaluating and comparing various detection algorithms. In various signal processing applications, the algorithm evaluation is performed by calculating the performance of simulated signals. However, the challenge is constructing simulated eye-tracking signals that can capture the disturbances and variations in raw signals to such an extent that they are helpful and authentic for performance evaluation. Moreover, due to the lack of a standard procedure for evaluating different event detection algorithms, it is not easy to compare the detection performances of various algorithms from different researchers [5].

In the past, researchers conducted a manual, time-consuming event detection. For example, ref. [8] devised a method to analyze eye movements at a rate of 10,000 s (almost three hours) of analysis time for 1 s of recorded data. Monty in [9] remarks that it is common to spend days processing data collected only in minutes. However, nowadays, event detection is almost exclusively done by applying a detection algorithm to the raw recorded eye-tracking data. For a long time, two broad classes of algorithms were used for eye movement event detection: The first class is the dispersion-based algorithms that detect fixations and assume the rest to be saccades [10]. The most well-known dispersion-based algorithm is the I-DT algorithm by Salvucci and Goldberg [11]. These algorithms detect the event by defining a spatial box that the raw recorded data must fit for a particular minimum time. The second class is the velocity-based algorithms that detect saccades and assume the rest to be fixations. The most well-known velocity-based algorithm is the I-VT algorithm [6,8]. These algorithms classify eye movements by calculating their velocity and comparing it to a predefined threshold.

The main contribution of this study is a comparison of different event detection algorithms on the same reference dataset. The study also summarizes the state-of-the-art in this field and compares the strengths and weaknesses of different algorithms. Various algorithms were compared from threshold-based, machine learning and deep learning domains. We used our own implementations of all the algorithms described in the literature with various parameters. Additionally, we developed and tested our own Convolutional Neural Network that can be used for event detection. All these implementations are available online in the form of Jupyter Notebooks (https://github.com/mebirtukan/EyeMovementEventDetectionAlgorithms, accessed on 1 June 2022). The paper’s outcome shows that correct eye movement event detection depends on many factors and thresholds that should always be considered when reporting popular eye movement recording parameters such as “average fixation duration” or “average saccade length”.

## 2. Eye Movement Events

**Figure 1:**
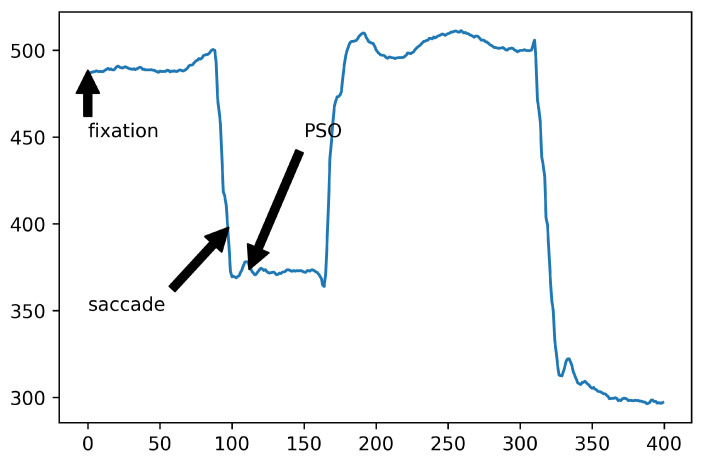
Graphical presentation of eye movement events for the horizontal axis.

As was already mentioned, raw eye movements are typically divided into events. In this section, we discuss different types of eye movement events. Eye-tracking signals do not only consist of different types of events of eye movement but also noise from different sources and blinks. Therefore, an event detection algorithm needs to consider such problems. The most often used event types are discussed further in the following subsections. An example is also shown in Figure 1.

Figure 1 shows an example of fixations, saccade and PSO in terms of position over time on the horizontal axis.

### 2.1. Fixations

A fixation is a movement when the eye is more or less still and focuses on an object. The purpose of the fixation movement is to stabilize the object on the fovea for clear vision. Fixation events may include three different types of distinct small movements: tremor, slow drift and microsaccades [7]. Tremor movement is a small wave-like eye motion with a frequency below 150 Hz and an amplitude around 0.01${}^{{}^{\circ}}$. The exact function of tremors still needs to be determined. Drift is a slow motion of the eye that co-occurs with tremor and it takes the eye away from the center of the fixation. A microsaccade is the fastest movement of the fixational eye movements, with a duration of about 25 ms. The role of a microsaccade movement is to quickly bring the eye back to its original position [7].

### 2.2. Saccades

A saccade is a rapid eye movement from one fixation point to another. A typical saccade has a duration between 30 and 80 ms and velocity between 30${}^{{}^{\circ}}$/s and 500${}^{{}^{\circ}}$/s [12]. There is a relationship between a saccade’s duration, amplitude and velocity. This relationship suggests that the larger saccades have larger velocities and last longer than the shorter ones [13]. The time from the onset of the stimulus to the initiation of the eye movement (called saccadic latency) is around 200 ms. It includes the time it takes for the central nervous system to determine whether a saccade should be initiated or not and, in this case, calculate the distance that the eye should move and transmit the neural pulses to the muscles that help to move the eyes [12]. Correct detection of saccades is essential because it is believed that a human brain does not “see” the image during the saccade. This phenomenon is called the saccadic suppression [14,15].

### 2.3. Smooth Pursuits

A smooth pursuit movement is performed when the eyes track a slowly moving object. It can only be performed when there is a moving object to follow. The latency of the smooth pursuit is around 100 ms and it is slightly shorter than the latency of saccadic movements [12]. It refers to the time it takes for the eye to start moving from the onset of the target object’s location. A smooth pursuit eye movement event can generally be divided into two stages: open-loop and closed-loop stages [16]. The initiation stage of the smooth pursuit is the pre-programmed open-loop stage, where the eye accelerates to catch up with the moving target. The closed-loop stage starts when the eye has caught up with the target and follows it with a velocity similar to that of the target object. In order to be able to follow the moving object in the closed-loop stage, the velocity of the moving object is estimated and compared to the velocity of the eye. If the velocity of the moving object and eye are different, for example, the eye lags behind the moving object, a catch-up saccade movement is performed to catch up with the object again. If the stimulus only consists of one moving target object that moves predictably, the eye will be able to follow it more accurately and with fewer catch-up saccades [16].

### 2.4. Post-Saccadic Oscillations

Rapid oscillatory movements that may occur immediately after the saccade are called post-saccadic oscillations (PSO). They can be described as oscillatory movements or instabilities that occur at the end of a saccade [16]. Post-saccadic oscillations are characterized by a slight wobbling movement that leads to fixation after a saccade. The cause of the PSO still needs to be clarified. Some researchers believe that it is caused by the recording device [17] and others believe the eye itself naturally wobbles after a saccade [12]. The PSOs are the type of eye movement for which there is typically the most substantial disagreement between manual raters. However, they are events that occur during recording eye movements and can influence the characteristics of fixations and saccades events [18]. PSOs are typically very short events with a duration of about 10–40 ms with an amplitude of 0.5–2${}^{{}^{\circ}}$ and velocities of 20–140${}^{{}^{\circ}}$/s [18].

### 2.5. Glissades

Another largely unexplored reason behind the variation in event detection results is the behavior of the eye at the end of many saccades, which indicates that the eye sometimes does not fix directly on the object but undershoots or overshoots it and then needs to do an additional corrective short saccade. Such an event is called a glissade. According to [19], glissades happen after about 50% of saccades, so they have a significant impact on the accurate measurement of saccade offset and onset of the subsequent fixation. Therefore, frequently the glissade is treated as a separate class of eye movement [20]. This movement is also known as a dynamic overshoot (rapid postsaccadic movement [21]) or a glissadic overshoot (slower postsaccadic movement [22]). Researchers have observed that glissades rarely occur simultaneously in both eyes [21]. Although frequently reported in the literature, it is only sometimes explicitly taken into account by event detection algorithms. Glissades are therefore treated unsystematically and differently across algorithms and even within the same algorithm; one glissade may be assigned to the saccade, whereas the next one is merged with the fixation [20].

## 3. Dataset

**Figure 2:**
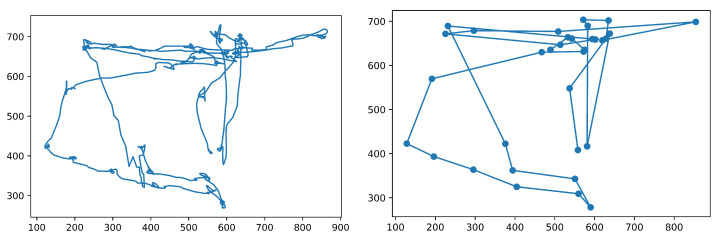
Example from one data file from the dataset. The (**left**) chart shows the raw gaze data, while the (**right**) one shows a sequence of fixations as annotated by one of the manual coders.

To test the performance of algorithms that we implemented and evaluated in this review paper, we used the publicly available dataset recorded with a Hi-Speed 1250 eye tracker from SensoMotoric Instruments (Teltow, Germany) at 500 Hz [23]. It is available online: https://github.com/richardandersson/EyeMovementDetectorEvaluation (accessed on 1 June 2022). The subjects were presented with static images, texts, video clips and simple moving dot stimuli. The data were manually labeled by two raters, Marcus Nyström (MN) and Richard Andersson (RA). It was annotated into fixation, saccades, PSOs, smooth pursuit, blinks and undefined. This study used image-viewing data labeled with fixations, saccades and post-saccadic oscillations. We tested and evaluated all the algorithms with the same dataset. One of the image-viewing sessions from the dataset in a raw format and divided into fixations is presented in Figure 2.

## 4. Classic Event Detection Methods

This section presents different eye movement event detection algorithms. Their performance was tested using a dataset discussed in Section 3.

There have been many works that have been done on developing eye movement event detection algorithms. The performance and adaptability of event detection algorithms depend on different factors, including the type of stimulus (i.e., static or dynamic), data quality (the data may be noisy), eye-tracking device (i.e., sampling frequency, binocular or monocular, fixed or mobile with rigid or flexible eye cameras). These differences make direct comparisons across methods and studies difficult. There are already several publications concerning eye movement event detection algorithm comparison. One of them is Andersson et al. [24]. In this paper, the authors evaluated and compared eye movement event detection algorithms and recommended the best method for future researchers. However, all the evaluated methods are threshold-based. Different methods detect different event types. For example, some methods identify fixation and saccade only, some identify fixation only and some identify fixation, saccade and PSO. Due to the difference in the event types that the algorithms identify, comparing an algorithm that detects single-class, binary-class and multi-class event classifiers is still unclear, because some methods can perform well for fixation and saccade classification and may perform poorly for other events. Another review of event detection algorithms was conducted by Gonca et al. [25]. They evaluated ten open-source threshold-based event detection algorithms.

This paper’s contribution over the above-mentioned publications is that we evaluated algorithms from threshold-based, machine learning and deep learning domains. We used different parameters and the same dataset to evaluate all the methods. The implemented event detection algorithms and their advantages and drawbacks are discussed below.

### 4.1. Manual Human Classification

In manual event classification, one or more human coders classify raw eye movement data into different event types based on subjective threshold values. Manual classification is still a common method for evaluating event detection algorithms and is treated as a “golden standard”. Manually classified data are frequently used as training data for machine learning algorithms. However, manual event classification is not an effective way to classify events. Firstly, it is time-consuming and secondly, different coders may use different subjective selection rules that give different results. For example, the authors of [26] used twelve experienced but untrained human coders to classify events in six minutes of eye-tracking data and found substantial differences between the classifications when average fixation duration and number of fixations were compared.

In this paper, we used the dataset annotated manually by two human coders, MN and RA, as discussed in Section 3 and we evaluated to what extent the two coders agreed to classify the same input data into events. We used the eye tracking data recorded during image viewing with the 4988 samples (UH21_img_Rome_labelled). Coder MN classified 4282 samples as fixations, 503 as saccades and 203 as PSOs. Coder RA labeled 4173 as fixations, 466 as saccades, 164 as PSOs, 177 as smooth pursuits and eight as undefined. The value of Cohen’s kappa was 90% and the confusion matrix between both coders is presented in Table 1. It shows that the classifications of the two agreed moderately. The most significant differences could be found in the PSO events with an F1-score as low as 85%. It seems that it is only a minor difference when only some samples between the end of the saccade and the onset of fixation are classified differently. However, such misclassification influences important parameters of eye movement data, like average fixation duration or average saccade length. Such parameters are frequently used in eye movement data analysis [27,28,29].

### 4.2. Dispersion Threshold-Based Event Detection Methods

Threshold-based methods are historically the first automated eye movement event classification algorithms and are still frequently used nowadays. The I-DT is the most straightforward and obvious eye movement event detection algorithm that classifies fixation points and saccade points based on the dispersion or spread distance of subsequent sample coordinates. The algorithm identifies gaze data as belonging to fixation when the samples are located within a spatially limited area (for example, 0.5${}^{{}^{\circ}}$) for minimum allowed fixation duration [30]. It follows that fixation points generally occur near one another. Saccades are then detected implicitly as everything else [11].

The algorithm requires two parameters to identify the events. These are *dispersion threshold* and *duration threshold*. The dispersion threshold can be set to 0.5 to 1${}^{{}^{\circ}}$ of visual angle if the distance from the eye to the screen is known. Otherwise, the dispersion threshold can be estimated from the exploratory analysis of data. The duration threshold is typically set to a value between 100 and 200 ms depending on task processing demands [31]. The algorithm calculates the dispersion of points in a window by simply summing the differences between the points’ maximum and minimum X and Y values, as shown in Equation (1).

(1) D=[max(x)−min(x)]+[max(y)−min(y)]

However, there are other methods of dispersion estimation methods discussed in [32]. The first method is distance dispersion, an algorithm that classifies every point as fixation if the distance between every point is no further than some threshold $D_{max}$. It is the most intuitive but less popular measure. Another method is the centroid-distance method, which requires the M of N points to be no further than some threshold $C_{max}$ from the centroid of N points. This algorithm has two versions, a consistent version that recomputes the distance of all points in the fixation to the centroid whenever the fixation is considered and a simpler (and faster) version that only checks the distance of the new point to be added.

The dispersion threshold methods exhibit poor performance detecting fixations and saccades when the signal is noisy [33]. Therefore, choosing the optimum threshold values is the most challenging step in the I-DT event detection algorithms. The impact of varying dispersion threshold values on the classification performance leads to biased results and misclassifications. For example, if the threshold value is set too high, false fixations might be identified and if it is set too low, actual fixations might be missed [34]. Due to this, parameter setting in the I-DT algorithms is crucial and may cause substantial differences in classification performance [34].

In this section, we evaluated the I-DT algorithm and the impact of threshold value on the classification performance was examined in a simple experiment. We used the dispersion threshold as a parameter. All input samples were converted into sequences containing the point and four points surrounding the classified point. The algorithm calculated dispersion for each sequence of points using Equation (1). We used the data collected from participants viewing images (see Section 3) and compared the results of the I-DT algorithm with different threshold values with the manual classification.

**Figure 3:**
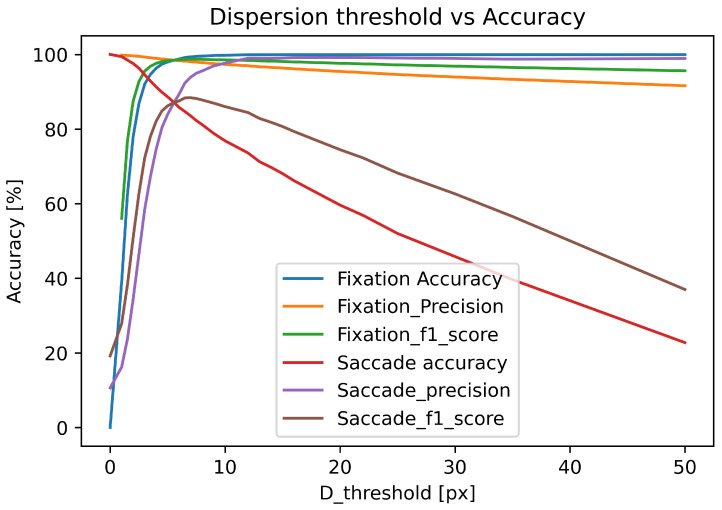
The accuracy for fixations and saccades of the I-DT algorithm for different dispersion thresholds.

The results are presented in Figure 3, which illustrates the impact of varying dispersion threshold value on the classification performance in the I-DT algorithm. The accuracy for each class is measured by recall, precision and F1-score from a confusion matrix. As shown from the results, the increase of the dispersion threshold value increases the fixation recall but, at the same time, decreases the saccade recall. On the other hand, increasing the threshold decreases fixation precision and increases saccade precision. The F1-score may be considered a good indicator of the correct threshold as it reaches the maximum value for both fixations and saccades for a similar threshold value.

For example, I-DT gives a maximum fixation recall of 99% and a minimum saccade recall of 82% at a dispersion threshold value of 7 px and a maximum saccade accuracy of 99% and a minimum fixation accuracy of 39% at the threshold value of 1 px. The optimum dispersion threshold value for the given example is 3.5 px. At this threshold value the I-DT gives 95% fixation recall value, 93% saccade recall, 98% fixation precision, 51% saccade precision, 96% fixation F1-score, saccade F1-score 66% and 0.6 Cohen’s kappa.

### 4.3. Velocity Threshold-Based Methods

The velocity threshold algorithm is another algorithm and the foundation for an automated/objective standard event detection algorithm. Many studies have adopted this approach [35,36]. It utilizes the fact that saccadic eye movements are characterized by higher velocity values than fixational movements. The velocity profiles of eye movements show essentially two velocity distributions: low velocities for fixations and high velocities for saccades. The I-VT method identifies events by calculating the point-to-point velocity and then classifies the event as fixation or saccade based on the value of this velocity [11]. The classic I-VT method is designed to classify all eye-tracking input data into fixations and saccades only. The other event types, such as smooth pursuits, post-saccadic oscillations and noises, are not considered.

**Figure 4:**
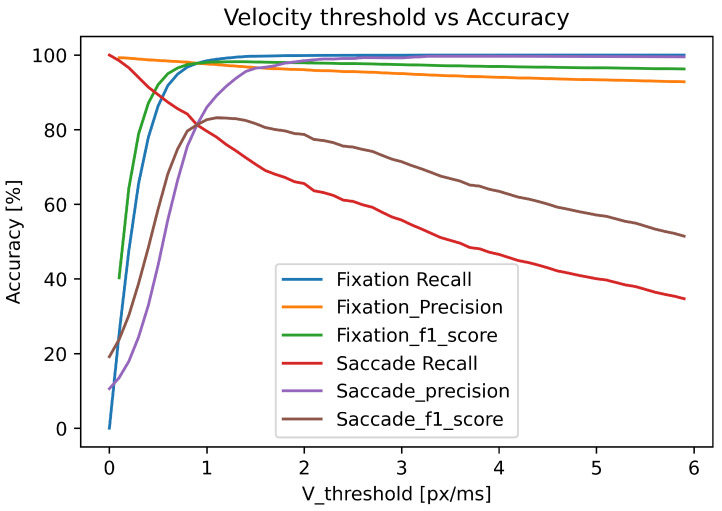
The accuracy for fixations and saccades of the I-VT algorithm for different velocity thresholds.

Figure 4 presents the impact of varying velocity thresholds on the classification performance of the I-VT algorithm. The classification accuracy of each class is measured by the recall, precision and F1-score calculated from the confusion matrix. Similarly to the I-DT algorithm, the increase in the velocity threshold increases the fixation classification recall and saccade precision while, at the same time, it decreases fixation precision and saccade recall.

In the given example, I-VT yields a maximum of 99% fixation recall at a threshold velocity of 3.5 px/ms and the saccade recall slightly decreases with the increase in velocity threshold value. The saccade recall reaches 98% and the fixation recall is 25% at the lowest velocity threshold value of 0.1 px/ms because, at this threshold value, most points are classified as saccades. Due to the impact of the threshold value on the classification accuracy of the I-VT algorithm, it is essential to find the optimum threshold value for both fixation and saccade accuracy. Therefore, in this case, the optimum velocity threshold value for I-VT is 0.5 px/ms. At this point, the fixation recall value is 92%, the saccade recall is 87%, the fixation precision is 96%, the saccade precision is 46%, the fixation F1-score is 94% and the saccade F1-score is 60%. The value of Cohen’s kappa at the optimum threshold value of 0.6 px/ms is only 0.5, which shows a moderate agreement between the human coders and I-VT classification algorithm.

The main drawback of the algorithm is that it uses only the velocity of the gaze without considering other possibilities like acceleration of the signal, direction of the gaze movement, the distance between the eye and camera, etc. It may result in misclassifications of events because the velocity ranges of the quickest slow eye movements and the slowest parts of saccades may overlap. Therefore, it seems that using other eye movement parameters such as acceleration, amplitude and position of eye movement could improve the results.

There is no standard optimum threshold velocity value and varying the threshold values affects the performance of the event detection algorithms. Due to these reasons, different researchers use different threshold values to develop and evaluate the performance of I-VT algorithms. Due to this variation, it is difficult to compare different studies of threshold-based event detection algorithms [11].

### 4.4. Fixation and Saccade Detection with the Presence of Smooth Pursuit

One of the main problems with the aforementioned I-DT and I-VT algorithms is that they do not take into consideration smooth pursuit events. In contrast, the automated classification methods proposed in [37] classify the eye movement data into fixations, saccades and smooth pursuits. The methods improve the existing event detection methods, I-VT and I-DT, by integrating both and adding one more threshold velocity. Reference [37] presents three possible algorithms, namely: IVVT, IVDT and IVMT algorithms.

The IVVT algorithm identifies fixations, saccades and smooth pursuits (SPs). First, it classifies fixations and saccades using the existing I-VT algorithm and then identifies SPs from fixations by adding one more threshold velocity.

The IVMP, first proposed by Javier San Agustin Lopez [38] and implemented by Oleg V. Komogortsev and Alex Karpov [37] classifies fixations and saccades by applying the I-VT algorithm and then distinguishes smooth pursuits from fixations using the movement pattern. As discussed in the I-VT-based classification method, the measured velocity can be used to classify gaze samples as fixations or saccades. However, as smooth pursuit movements can have similar velocities to fixations, the simple velocity method cannot be used to differentiate smooth pursuits from fixations. In order to determine whether the eye is performing a fixation or a smooth pursuit movement, the direction of movement is analyzed in a temporal window with a size of $T_{w}$. In that window, the magnitude of movement is computed by analyzing angles created by every pair of adjacent positional points and the horizontal coordinate axis. Then, the magnitude of the movement is compared with threshold movement ($T_{m}$). If the magnitude of movement is above the threshold value, it is marked as smooth pursuit and if it is below the threshold value, it is marked as a fixation.

The IVDT algorithm uses both I-VT and I-DT to classify fixations, saccades and smooth pursuits. As in IVVT, it first applies the velocity threshold $V_{T}$ to classify saccades and fixations and classifies the point as a saccade if the velocity is above the $V_{T}$. Then the dispersion threshold $D_{T}$ is applied to identify the rest of the data into fixations and smooth pursuits.

The working principle of the IVMP algorithm is the same as discussed above. It uses $V_{T}$ and movement pattern to classify events as fixation, saccade or smooth pursuit. At first, it applies the $V_{T}$ to classify all data into fixations and saccades and then movement pattern $T_{m}$ to distinguish smooth pursuits from fixations.

It is possible to introduce qualitative and quantitative behavior scores to calculate optimal threshold values for each algorithm. However, these scores are data driven and may differ for data obtained from different eye trackers. Therefore, finding the optimum threshold values still needs to be solved.

### 4.5. Automated Velocity Threshold Data Driven Event Classification Method

The main problem in the previously discussed approaches was finding the correct threshold—which is especially difficult in a noisy signal. Therefore, an automated velocity threshold data driven event classification method was proposed [20]. The algorithm is able to adaptively find the threshold and avoid the influence of noise. Additionally, it identifies the glissades as separate event types. It is designed to overcome the noise sensitivity that occurs in previous algorithms by designing adaptive $V_{T}$ values considering different levels of noise occurrence. It removes noises and unwanted variations by calculating velocity and acceleration profiles that the previous works calculated using simple sample-to-sample subtraction. However, the outcome of this calculation is noisy. Therefore, the automated data driven method eliminates noise by calculating velocity and acceleration based on Duchowski et al. [39] who calculate velocity and acceleration based on finite impulse response (FIR) by using filters.

The drawback of this method is that the glissade is detected based on duration only. This means that it occurs in half the saccade duration [20]. So, the saccade with a short duration may be classified as a glissade and the glissade with a long duration may be classified as a saccade, since there is no other parameter to distinguish glissades from saccades. The algorithm is designed to detect glissades with the presence of fixation and saccade only and it does not consider other events like SP and PSO. It also cannot deal with glissade-like movements preceding a saccade.

## 5. Machine Learning-Based Event Detection Methods

The major drawback of all threshold-based event detection algorithms is that the user is left with a number of parameters that have to be adjusted based on eye movement data quality and finding the optimum threshold values is challenging. Another drawback is that the threshold-based algorithms are designed to solve a specific problem in one-step classification (like fixation and saccade). Eye movement event classification using machine learning addresses these problems [40]. Machine learning algorithms classify raw eye-tracking data into event types without manually setting any parameters and calculating and finding threshold values. They learn the correct classification based on some training data.

Typically, for most machine learning algorithms, it is assumed that the classification of one specific gaze point to the event depends on the point’s neighborhood. Therefore, the standard input to the model is a set of properties from some number of gaze points before and (for off-line classification) some number of gaze points after the point is classified. These properties may be just raw coordinates, but frequently properties such as velocity, acceleration, movement direction or jerk are also used. The window size is one of the basic parameters for every model.

We have evaluated and discussed some event detection methods using machine learning algorithms. Two models utilizing Random Forest classifier and Convolutional Neural Networks are implemented and evaluated with the same dataset.

### 5.1. Event Classification Using Random Forest Classifier

Fully automated eye movement event classification using a Random Forest classifier was first proposed in [41] to classify fixations, saccades and post-saccadic oscillations. Classification performance was compared with the current state-of-the-art algorithms and manual human coders. The paper stated that the machine learning algorithm outperforms the current state-of-the-art algorithms and almost reaches the performance of manual human experts. However, this performance was only achieved for high-quality data (with low noise levels). In this section, we describe our own implementation of the algorithm that utilizes the Random Forest classification model for event classification.

We implemented the Random Forest classification algorithm to classify eye-tracking data into fixations, saccades and PSOs. We evaluated the classification performance regarding fixation classification accuracy, saccade accuracy and PSO classification accuracy. This algorithm can detect eye movements in the continuous gaze stream and assign labels for all three eye movement types simultaneously. We, therefore, further evaluated the algorithm’s classification performance separately for the three-class detection problem by evaluating sample-by-sample predictions, confusion matrices and finally, by evaluating the classification performance of each class.

To build the model, we used velocity as a parameter: we converted eye tracker data coordinate points into the velocity domain and created sequences of samples with a sequence length of 40. We also tested shorter and longer sequences, but this did not significantly impact the result. Therefore, the input to the model was a sequence of gaze samples of size 40×2.

**Figure 5:**
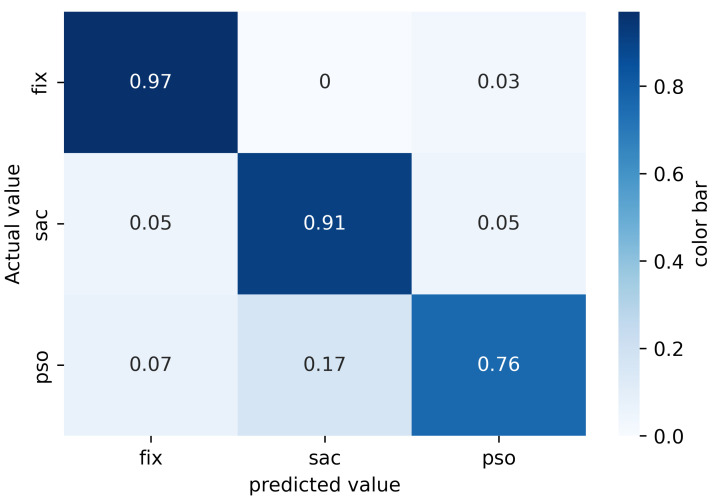
The RF confusion matrix.

Figure 5 shows the confusion matrix for the sample-by-sample evaluation. Fixations are labeled correctly in 97% of cases, while for PSO and saccades there are tendencies to be labeled as fixations. Saccade and PSO are correctly identified in 91% and 76% of frames, respectively. About 7% of the PSOs are falsely classified as fixation and 17% of PSOs are classified as a saccade. This happens because most of the training events were fixations and the model naturally tends to classify all ambiguous samples as fixations.

Table 2 summarizes the performance of the RF classifier in terms of accuracy, precision, recall and F1 score for each class. The results show that the classification of PSO events is the most challenging.

### 5.2. Using Convolutional Neural Networks

Convolutional Neural Networks are good at finding patterns in data, so it is possible to use them in eye movement event detection. One example of such an application is the method proposed in [42], which is based on the deep Convolutional Neural Network that, for each sample, predicts a sequence of probabilities of belonging to a fixation, saccade, or smooth pursuit from a sequence of gaze samples. The method tries to address the drawback of previous methods, which use signal shape and amplitude to determine or to classify the eye movement events, which may be problematic, for instance, for smooth pursuits. The proposed method uses the signal’s frequency to classify the data into event types. That means it first converts the raw gaze data into the frequency domain of the raw signal using Fast Fourier Transform (FFT) and then passes the frequency representation of the signal to the CNN network, which in turn gives the output of a three-dimensional activation signal. Each signal represents the probability of each eye movement type (fixation, saccade and SP). Finally, the label with a high probability is assigned to the central sample in the window.

The method is not end-to-end, as the input to the network is the FFT output. It uses hand-crafted features—input data that need to be transformed into the frequency domain. The proposed method classifies fixations, saccades and smooth pursuits without considering other events like PSO. The method outperforms the old algorithms based on simple dispersion and velocity thresholding.

**Figure 6:**
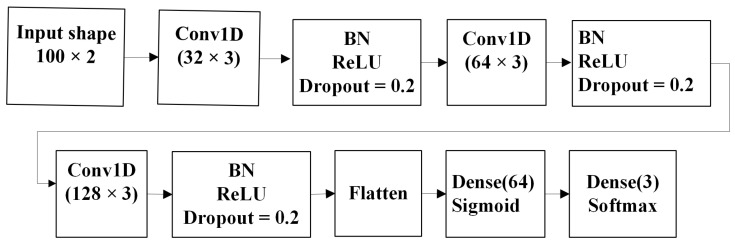
The architecture of the CNN used in the experiment.

To test the ability of the CNN network to classify eye movement events, we created a simple network presented in Figure 6. The network takes a continuous stream of two-dimensional gaze samples as input. To obtain a prediction for each gaze sample, the window moves over the sequence one by one. We first convert the x and y coordinate data into horizontal and vertical velocity components by calculating sample-to-sample velocity. To obtain relevant eye movement characteristics, the stream of gaze samples is analyzed in windows of 100 samples which gives the best results in our experiments.

The network is composed of different layers, precisely three convolutional layers with a gradually increasing number of filters (32, 64 and 128) with a kernel size of 3, a batch normalization operation before activation and an output layer. Input to the network is a sequence of gaze samples of shape 100 × 2. The network architecture is shown in Figure 6.

**Figure 7:**
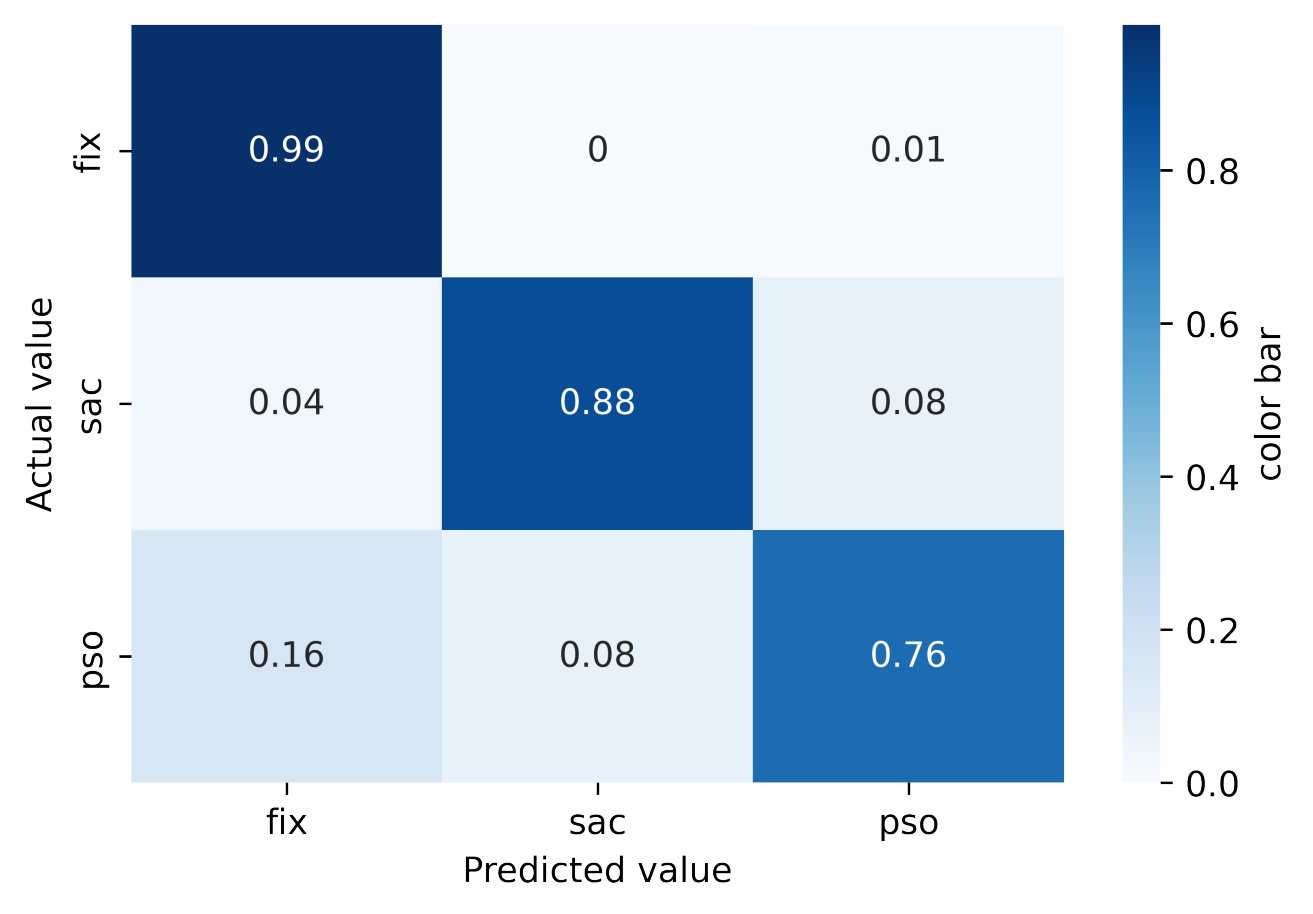
Confusion matrix for the CNN Classifier.

Figure 7 shows the confusion matrix for the CNN classification. Fixations are correctly classified in 99% of the cases; saccades are correctly classified in 88% and PSOs are correctly classified in 76% of the cases. A total of 4% of the saccades are falsely classified as fixations and 16% of PSOs are falsely classified as fixations. Additionally, 8% of saccades are classified as PSOs. The classification results shows that CNN performs well for fixation and saccade classification. However, the classification performance for the PSOs is far from perfect.

Table 3 summarizes the performance of the CNN classifier in terms of accuracy for each class, precision, recall and F1 score. The results show that—similarly to the RF-based algorithm—the best scores are reached for fixations and the worst for PSOs.

### 5.3. Using Recurrent Neural Networks

Eye movement recordings form a time series, so it is natural that algorithms proven to operate well on time series could be used for event classification. One of the possibilities is to use Recurrent Neural Networks.

The paper [43] presents an excellent example of such an application. It presents the network that classifies the raw eye movement data into fixations, saccades and smooth pursuits. The network is a combination of the 1D-convolutional network and the BLSTM layer (a classic recurrent layer that preserves information about previous samples). It is built of a one-dimensional temporal convolutional network with one time-distributed dense layer both before and after the BLSTM. Individual feature sets for the model are raw XY coordinates, speed, direction and acceleration. However, the method exhibits poor performance when it takes a combination of parameters. Researchers used a publicly available manually annotated eye-tracking dataset with over four hours of 250 Hz low-frequency recordings done with SR Research EyeLink II and 500 Hz recordings done with SensoMotoric Instruments Hi-Speed 1250 eye tracker. The algorithm is evaluated only by a clean and manually labeled dataset. Validating the algorithm with raw eye movement data is recommended to evaluate the actual algorithm’s performance. The combination of direction and speed showed a noticeable improvement over using them separately. Acceleration as an additional feature did not improve average detection performance, probably due to its inability to distinguish smooth pursuits from fixations.

## 6. Comparison

The purpose of this paper was to compare different eye movement event detection algorithms. This was done by evaluating the performance of four different event classification algorithms from the domain of threshold-based, machine learning-based and deep learning algorithms as well as the mutual performance of two human evaluators.

Each row in Table 4 shows the performance evaluation metrics for each event class and the columns show the classification algorithms. The results show that I-DT performs better than I-VT in all performance-measuring metrics. However, both RF and CNN algorithms outperform the threshold-based algorithms (I-VT and I-DT) in terms of all performance-measuring metrics except saccade recall. In the case of RF and CNN classification models, there is no significant difference for fixation and saccade classification. However, CNN outperforms RF in PSO precision, F1-score and Cohen’s kappa. Table 5 summarizes strengths and weaknesses of all implemented algorithms.

**Figure 8:**
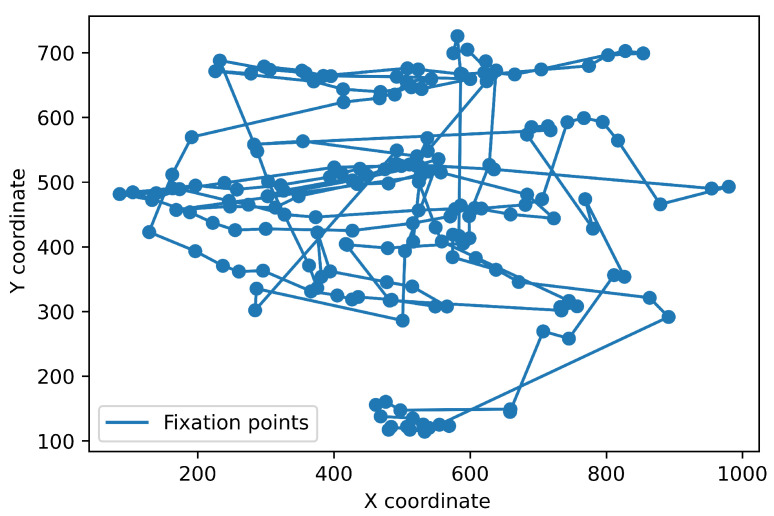
Eye fixations obtained from the I-VT algorithm at optimum threshold value of 3.5 px/ms. It is visible that many fixations occur nearby and could probably be combined together.

**Figure 9:**
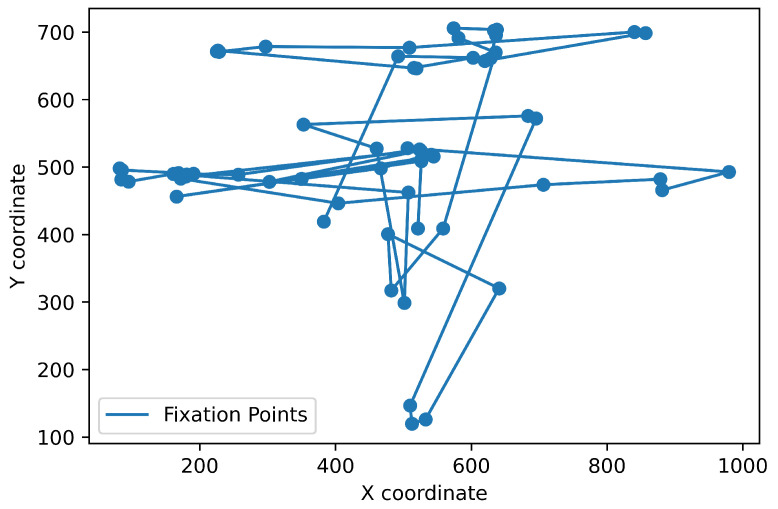
Eye fixations obtained from the RF algorithm. Compared to Figure 8, there are far fewer fixations.

It should be emphasized that the presented result takes into account only point-to-point comparisons, so each gaze point is classified as a part of the specific event. In fact, the event itself always takes some time. For instance, the average fixation duration should be about 250 ms [44]. Therefore, the following typical step in event detection is converting a sequence of subsequent points classified as fixations into one fixation with the location calculated as the median of these points’ locations. If there is a gap between fixation sequences (several points classified differently), two sequences are classified as two separate fixations. Obviously, this significantly impacts specific measures like the overall number of fixations and average fixation duration. Therefore, we compared the obtained results after merging subsequent points. The results for I-VT and RF are presented in Figure 8 and Figure 9, respectively. It occurred that the I-VT algorithm found 189 fixations with an average duration of 121 ms while the RF algorithm found only 64 fixations with an average duration of 264 ms. Considering that the manual coder found 91 fixations with average duration 222 ms, it may be concluded that threshold-based algorithms require the additional step of merging subsequent fixations that are located nearby (hence: additional threshold parameters). In contrast, machine learning algorithms deal with this problem internally. It is clearly visible in Figure 8 and Figure 9.

## 7. Conclusions

In this study, we evaluated event detection algorithms from different domains: the I-VT and I-DT from the threshold-based domain, the Random Forest model from machine learning and the CNN model from deep learning domains. We compared their classification performance by using the same dataset for all methods. The agreement between human coders and algorithms was also evaluated. The impact of varying threshold values on the classification performance of threshold-based algorithms was discussed. The results revealed that threshold values critically affect the classification results of the I-VT and I-DT algorithms. Due to this, finding the optimum threshold is challenging in threshold-based algorithms. The RF and CNN algorithms outperform threshold-based algorithms in all performance-measuring metrics and can perform multi-class classification.

This work evaluated event detection algorithms to classify event data into fixations, saccades and PSOs. We did not consider smooth pursuit (SP) events. We used only these three event types because we used only velocity parameters in the algorithms to classify events. More than a velocity value is needed to differentiate the SP from fixations due to their similar behavior in terms of velocity. Therefore, during our future research, we plan to extend the classification by incorporating smooth pursuits and other event types. Additionally, we would like to analyze the usability of other eye movement signal parameters like acceleration, jerk and frequency domains.

The code in Python for data preparation and all performed classifications will be available upon the acceptance of the paper.

## Figures and Tables

**Table 1:** Confusion matrix between two manual coders.

RA\MN	Fixation	Saccade	PSO
fixation	4111	4	54
saccade	28	444	10
PSO	26	14	297

**Table 2:** Each class classification performance with RF classifier.

Classes	Accuracy	Precision	Recall	F1-Score
Fixation	97%	99%	97%	98%
Saccade	92%	87%	91%	89%
PSO	76%	64%	76%	69%

**Table 3:** Each class classification performance with CNN classifier.

Classes	Accuracy	Precison	Recall	F1-Score
Fixation	99%	98%	99%	99%
Saccade	89%	93%	89%	91%
PSO	75%	83%	75%	79%

**Table 4:** Comparison of the classification performance of the algorithms.

Performance Metrics	IVT [11]	IDT [11]	RF	CNN	Coder MN [26]	Coder RA [26]
Fixation Accuracy	92%	95%	97%	99%	99%	99%
Saccade Accuracy	87%	93%	92%	89%	92%	96%
PSO Accuracy	-	-	76%	75%	88%	82%
Fixation F1-score	94%	96%	99%	99%	99%	99%
Saccade F1-score	60%	66%	87%	91%	94%	94%
PSO F1-Score	-	-	64%	79%	85%	85%
Fixation Recall	92%	95%	97%	99%	99%	99%
Saccade Recall	87%	93%	92%	89%	92%	96%
PSO Recall	-	-	76%	75%	88%	82%
Fixation Precision	96%	98%	99%	98%	99%	99%
Saccade Precision	46%	51%	87%	93%	96%	92%
PSO precision	-	-	64%	83%	82%	88%
Cochen’s Kappa	0.5	0.6	0.83	0.88	1	0.90

**Table 5:** Strengths and weaknesses of event detection algorithms.

Algorithms	Strengths	Weaknesses
Human Coders [26]	Manual coding is still a common method for evaluating event detection algorithms and manually classified data are used as training data for machine learning algorithms.	Time consuming, different coders may use different subjective selection rules that give different results because parameters and threshold values are set manually by the coder.
I-VT [11]	Simple to implement and understand. Uses one threshold value which is velocity to identify events from raw input data. Performs very well for fixation and saccade identification in single identification step. Low computational resources.	Although it is simple, I-VT is rarely used in real implementations. It is sensitive to noisy signals with many outliers. Finding optimum threshold value is challenging as there is no standard optimum threshold value. Identifies fixations and saccades only.
I-DT [11]	The first automated event detection algorithm. Performs fixation and saccade identification with human level identification performance. I-DT is frequently available in commercial software.	Performance is affected by choice of threshold values. Choosing a dispersion calculation method is challenging as different dispersion calculation methods affect the dispersion value. Designed for fixation and saccade identification only.
RF	No threshold value is needed. Performs multi-class classifications so may be used for various events. It is a fully automated event classification method. Performs fixation and saccade identification with human level performance.	Requires a significant amount of correctly annotated data for training. In our implementation only velocity features were used to identify events as fixation, saccade and PSO. The classification result for PSO was because of misclassification between saccade and PSO due to the similarity of saccade and PSO in terms of velocity.
CNN	Like RF, CNN also addresses threshold-based detection method problems. Performs single step end-to-end detection without human intervention. Performs at human level detection for fixation identification.	Requires for training even more correctly annotated data than the RF algorithm. We used only velocity parameters to identify events from input data. Smooth pursuit was not considered because the velocity parameter that we used is not sufficient to identify smooth pursuit from fixation as both of them are low velocity movement types. Other parameters such as direction or movement patterns should be used to identify smooth pursuit. CNN performed worse than RF and I-DT for saccade detection.

## Data Availability

The code presented in the paper is available at https://github.com/mebirtukan/EyeMovementEventDetectionAlgorithms, accessed on 1 June 2022.
